# Supplementary material for: Deviation from the matching law reflects an optimal strategy involving learning over multiple timescales
Source: Nat Commun. 2019 Apr 1;10:1466. doi: 10.1038/s41467-019-09388-3 (PMC6443814; doi:10.1038/s41467-019-09388-3)
Supplement: Supplementary file 1 — Supplementary Information [file 41467_2019_9388_MOESM1_ESM.pdf]

Supplementary Notes

Probability estimation of a binary sequence over blocks of trials

Here we illustrate our analytical results of the probability estimation by a model that learns inputs over multiple timescales. For this, consider the following statistical estimation task. An observer (a monkey statistician) observes the time series, s_t, of the (conditionally) independent drops of a coin (heads $s = 1$, tails $s = 0$), where the head probability, p, is constant over each block of T coin drops (trials), while the probability changes on the first trial of each block to a new p, by independently sampled from a distribution, $\pi(\cdot)$, on the interval $[0, 1]$ (For now, our default choice will be the uniform distribution on $[0, 1]$). The observer's goal is to estimate the current head probability, p, by collecting/integrating the observations over trials, over a flexible combination of two, fixed, time scales. More precisely the current estimate, v_t, of p, at time t, is given by

$$v_t = (1 - w_{Slow})v_{1,t} + w_{Slow}v_{2,t} \tag{11}$$

where $0 \leq w_{Slow} \leq 1$, and $v_{1,t}$ and $v_{2,t}$ are leaky integrations of the recent history of s_t over the time scales τ_1 (fast) and τ_2 (slow), respectively, i.e.

$$v_{i,t} = (1 - q_i)v_{i,t-1} + q_i s_t \tag{12}$$

where the learning rates, q_i, are defined by the inverse of time constants $1/\tau_i$. Solving 12 (in steady state, i.e. after many blocks) we have

$$v_{i,t} = q_i \sum_{n=0}^{\infty}(1 - q_i)^n s_{t-n}. \tag{13}$$

We assume that the time-scales, q_1 and q_2, are fixed, perhaps reflecting a form of hardware constraints, but we consider w_{Slow} to be flexible. We want to find the optimal w_{Slow} leading to the minimum possible average square error for the estimator v_t, i.e. the w_{Slow} that minimizes the long-time average of $(v_t - p)^2$, given the knowledge of block size T and the internal time-scales, τ_i.

We will adopt the following index notation. We use t as the trial index, n as the trial lag (into the past), and k as the block index. We denote the current block by $k = 0$, with $k = 1, 2, \ldots$ indicating past blocks (so $k > 0$ indicates the block-lag into the past). Thus p_0 is the head probability of the current block, p_1 that of the previous block, and so on. We choose the time origin such that the first trial of the current block ($k = 0$) has $t = 1$, with trials in past blocks having zero or negative t's.

We also adopt the following averaging notations. We denote the average of a quantity, conditional on knowing the full sequence of block-probabilities $p_{0:\infty}$, by $\langle\cdot\rangle$, i.e.

$$\langle X_t \rangle \equiv \mathbb{E}[X_t | p_{0:\infty}]. \tag{14}$$

We indicate averaging over $p_{0:\infty}$ by $[\cdot]_\pi$, i.e.

$$[X]_\pi \equiv \int X_t(p_{0:\infty}) \prod_{k=0}^{\infty} d\pi(p_k). \tag{15}$$

Finally we indicate averaging over the duration of a block by a bar:

$$\overline{X} \equiv \frac{1}{T}\sum_{t=1}^{T} X_t \tag{16}$$

Thus we set out to calculate the long-run average square error, which in the above notation is given by

$$\overline{[\langle (v_t - p_0)^2 \rangle]_\pi} \tag{17}$$

and then find the optimal w_{Slow} that minimizes this cost.

We start by evaluating $\left\langle (v_t - p_0)^2 \right\rangle$ which can be decomposed in the standard way, into the variance of the estimator and its squared bias, i.e.

$$\begin{aligned} \left\langle (v_t - p_0)^2 \right\rangle &= \left\langle (v_t - \langle v_t \rangle)^2 \right\rangle + (\langle v_t \rangle - p_0)^2 && (18) \\ &= \left\langle \delta v_t^2 \right\rangle + (\langle v_t \rangle - p_0)^2. && (19) \end{aligned}$$

In model-fitting and parameter estimation, variance generally quantifies the degree to which an estimator is sensitive to noise in the data (the noise in $\{s_t\}$, in our case), in other words it quantifies how much it can fit noise, i.e. over-fit, while the bias normally quantifies how rigid or inflexible a model is (its inability to conform to and capture certain aspects of the data, hence creating biases). Generally speaking, the more complex a model, the more flexible it is, and the lower is its bias and higher its variance (it is prone to over-fitting). One way of reducing the variance and estimator-flexibility is to introduce a prior (which could represent past experience), making the estimator not rely entirely on currently observed data but also on prior knowledge (or past experience). In our case, the integrator with the slow time-scale, $v_{2,t}$ serves this purpose; it represents the prior knowledge acquired over long time scales (previous blocks), and is less altered by observations in the current block (it has a smaller learning rate). But by that virtue it is less flexible and retains its bias longer and is slow in adapting to the present value of p in the current block.

To summarize

- bias = prejudice = rigidity = low model complexity = slow time scale,
- variance = open-mindedness = flexibility = high model complexity = fast time scale.

A good model/observer must balance the two.

Variance

We will first look at the long-run average variance $\overline{[\langle \delta v_t^2 \rangle]_\pi}$. From Eq. (11), the conditional variance is given by

$$\left\langle \delta v_t^2 \right\rangle = (1 - w_{Slow})^2 \left\langle \delta v_{1,t}^2 \right\rangle + w_{Slow}^2 \left\langle \delta v_{2,t}^2 \right\rangle + 2w_{Slow}(1 - w_{Slow}) \left\langle \delta v_{1,t} \delta v_{2,t} \right\rangle \tag{20}$$

where $\delta v_{i,t} \equiv v_{i,t} - \langle v_{i,t} \rangle$. From Eq. (13), $v_{i,t}$ is a linear combination of independent random variables, s_t (the latter are independent only when conditioning/fixing $p_{0:\infty}$), thus its variance is the sum of the variances of the terms in this sum. The variance of s_{t-n} in block k is given by

$$\left\langle \delta s_{t-n}^2 \right\rangle = p_k(1-p_k), \qquad k = -\left\lfloor \frac{t-n-1}{T} \right\rfloor \equiv k(t-n). \tag{21}$$

Thus

$$\left\langle \delta v_{i,t}^2 \right\rangle = q_i^2 \sum_{n=0}^{\infty} (1-q_i)^{2n}\; p_{k(t-n)}(1-p_{k(t-n)}). \tag{22}$$

Similarly

$$\langle \delta v_{1,t} \delta v_{2,t} \rangle = q_1 q_2 \sum_{n=0}^{\infty} (1-Q)^n\; p_{k(t-n)}(1-p_{k(t-n)}), \tag{23}$$

where we defined

$$1 - Q \equiv (1-q_1)(1-q_2). \tag{24}$$

Since $[p_k(1-p_k)]_\pi$ is the same in all blocks, hence independent of $k(t-n)$, we can readily calculate the variance averaged over $p_{0:\infty}$, by summing the infinite geometric series, obtaining

$$\left[\left\langle \delta v_{i,t}^2 \right\rangle\right]_\pi = \frac{1}{2\tau_i - 1} \left[p(1-p)\right]_\pi , \tag{25}$$

$$\left[\langle \delta v_{1,t} \delta v_{2,t} \rangle\right]_\pi = \frac{1}{\tau_1 + \tau_2 - 1} \left[p(1-p)\right]_\pi . \tag{26}$$

where we used $q_i^2 \sum_{n=0}^{\infty} (1-q_i)^{2n} = \frac{q_i^2}{1-(1-q_i)^2} = \frac{q_i}{2-q_i} = \frac{1}{2\tau_i - 1}$ and $q_1 q_2 \sum_{n=0}^{\infty} (1-Q)^n = \frac{q_1 q_2}{1-(1-Q)} = \frac{q_1 q_2}{q_1+q_2-q_1 q_2} = \frac{1}{\tau_1+\tau_2-1}$. In particular, $\left[\left\langle \delta v_t^2 \right\rangle\right]_\pi$ is time independent:

$$\left[\left\langle \delta v_t^2 \right\rangle\right]_\pi = \overline{\left[\left\langle \delta v_t^2 \right\rangle\right]_\pi} = [p(1-p)]_\pi \left[\frac{(1-w_{Slow})^2}{2\tau_1 - 1} + \frac{w_{Slow}^2}{2\tau_2 - 1} + \frac{2 w_{Slow}(1-w_{Slow})}{\tau_1 + \tau_2 - 1} \right]. \tag{27}$$

Variance conditional on p_0: transient behavior

For completeness, we will also calculate the variance conditional on p_0 as well, obtaining its full transient behavior throughout the block. That is, here we will only average over $p_{1:\infty}$, but not over t and p_0. Going back to Eq. (22), we rewrite it by decomposing the sum into sums over blocks:

$$\left\langle \delta v_{i,t}^2 \right\rangle = q_i^2 \left\{ \sum_{n=0}^{t-1} (1-q_i)^{2n} p_0(1-p_0) + \sum_{k=1}^{\infty} p_k(1-p_k) \sum_{n=t+(k-1)T}^{t+kT-1} (1-q_i)^{2n} \right\} \tag{28}$$

It helps to rewrite this in the form

$$\left\langle \delta v_{i,t}^2 \right\rangle = q_i^2 \left\{ \sum_{n=0}^{\infty} (1-q_i)^{2n} p_0(1-p_0) + \sum_{k=1}^{\infty} \left[p_k(1-p_k) - p_0(1-p_0)\right] \sum_{n=t+(k-1)T}^{t+kT-1} (1-q_i)^{2n} \right\} \tag{29}$$

where the first term pretends that the probability was p_0 in the entire past, and the second term corrects for this by adding the difference of the variances accumulated over previous blocks contributed by the true probability, p_k, and the current one, p_0, respectively. By the geometric series formula the sum over block k is given by

$$\sum_{n=t+(k-1)T}^{t+kT-1}(1-q_i)^{2n} = (1-q_i)^{2t+2(k-1)T}\sum_{n=0}^{T-1}(1-q_i)^{2n} \tag{30}$$

$$= (1-q_i)^{2t+2(k-1)T}\frac{1-(1-q_i)^{2T}}{1-(1-q_i)^2} \tag{31}$$

$$= (1-q_i)^{2t}\frac{1-(1-q_i)^{2T}}{1-(1-q_i)^2}(1-q_i)^{2T(k-1)}. \tag{32}$$

Using $\frac{q_i^2}{1-(1-q_i)^2} = \frac{q_i}{2-q_i} = \frac{1}{2\tau_i - 1}$, we then have

$$\langle \delta v_{i,t}^2 \rangle = \frac{1}{2\tau_i - 1}\left\{p_0(1-p_0) + (1-q_i)^{2t}\left(1-(1-q_i)^{2T}\right)\sum_{k=0}^{\infty}(1-q_i)^{2Tk}\left[p_{k+1}(1-p_{k+1}) - p_0(1-p_0)\right]\right\}$$

$$= \frac{1}{2\tau_i - 1}\left\{p_0(1-p_0) + (1-q_i)^{2t}\frac{\sum_{k=0}^{\infty}(1-q_i)^{2Tk}\left[p_{k+1}(1-p_{k+1}) - p_0(1-p_0)\right]}{\sum_{k=0}^{\infty}(1-q_i)^{2Tk}}\right\} \tag{33}$$

Similarly for $\langle \delta v_{1,t}\delta v_{2,t}\rangle$ we have

$$\langle \delta v_{1,t}\delta v_{2,t}\rangle = q_1 q_2\left\{\sum_{n=0}^{\infty}(1-Q)^n p_0(1-p_0) + \sum_{k=1}^{\infty}\left[p_k(1-p_k) - p_0(1-p_0)\right]\sum_{n=t+(k-1)T}^{t+kT-1}(1-Q)^n\right\} \tag{34}$$

$$= \frac{1}{\tau_1+\tau_2-1}\left\{p_0(1-p_0) + (1-Q)^t\left(1-(1-Q)^T\right)\sum_{k=0}^{\infty}(1-Q)^{Tk}\left[p_{k+1}(1-p_{k+1}) - p_0(1-p_0)\right]\right\}$$

$$= \frac{1}{\tau_1+\tau_2-1}\left\{p_0(1-p_0) + (1-Q)^t\frac{\sum_{k=0}^{\infty}(1-Q)^{Tk}\left[p_{k+1}(1-p_{k+1}) - p_0(1-p_0)\right]}{\sum_{k=0}^{\infty}(1-Q)^{Tk}}\right\}$$

where we used $\frac{q_1 q_2}{1-(1-Q)} = \frac{q_1 q_2}{q_1+q_2-q_1 q_2} = \frac{1}{\tau_1+\tau_2-1}$. Averaging over $p_{1:\infty}$ and summing the infinite geometric series over blocks, combining contributions as in Eq. (20), and using Eq. (27), we then obtain

$$\left[\langle \delta v_t^2 \rangle | p_0\right]_\pi = \frac{p_0(1-p_0)}{\left[p(1-p)\right]_\pi}\left[\langle \delta v_t^2 \rangle\right]_\pi \tag{35}$$

$$+\left(\left[p(1-p)\right]_\pi - p_0(1-p_0)\right)\times \tag{36}$$

$$\left[(1-q_1)^{2t}\frac{(1-w_{Slow})^2}{2\tau_1 - 1} + (1-q_2)^{2t}\frac{w_{Slow}^2}{2\tau_2 - 1} + (1-q_1)^t(1-q_2)^t\frac{2w_{Slow}(1-w_{Slow})}{\tau_1+\tau_2-1}\right]$$

Here, the first line gives the steady state value of the variance in the current block if it was infinitely long, and the second and the third line gives the transient memory of variance from previous trials, which wears off for $t \gg \tau_2$. It starts from a value equal to the average variance $\left[\langle \delta v_t^2 \rangle\right]_\pi = \left[p(1-p)\right]_\pi\left[\frac{(1-w_{Slow})^2}{2\tau_1-1} + \frac{w_{Slow}^2}{2\tau_2-1} + \frac{2w_{Slow}(1-w_{Slow})}{\tau_1+\tau_2-1}\right]$ at $t = 0$ and eventually (given an infinitely long current block) relaxes to its steady-state value based on the current p_0 as opposed to the average, i.e. to $p_0(1-p_0)\left[\frac{(1-w_{Slow})^2}{2\tau_1-1} + \frac{w_{Slow}^2}{2\tau_2-1} + \frac{2w_{Slow}(1-w_{Slow})}{\tau_1+\tau_2-1}\right]$.

Squared bias

First let us calculate $\langle v_{i,t}\rangle$. Using the notation of Eq. (21), from Eq. (13) we have

$$\langle v_{i,t}\rangle = q_i \sum_{n=0}^{\infty}(1-q_i)^n \; p_{k(t-n)} \tag{37}$$

Again we can decompose this over blocks:

$$\langle v_{i,t}\rangle = q_i \left\{ \sum_{n=0}^{t-1}(1-q_i)^n p_0 + \sum_{k=1}^{\infty} p_k \sum_{n=t+(k-1)T}^{t+kT-1} (1-q_i)^n \right\} \tag{38}$$

and again it helps to rewrite this as

$$\langle v_{i,t}\rangle \;=\; q_i \left\{ \sum_{n=0}^{\infty}(1-q_i)^n p_0 + \sum_{k=1}^{\infty}(p_k - p_0) \sum_{n=t+(k-1)T}^{t+kT-1} (1-q_i)^n \right\}. \tag{39}$$

Noting that $q_i \sum_{n=0}^{\infty}(1-q_i)^n = 1$, for the bias component, $b_{i,t} \equiv \langle v_{i,t}\rangle - p_0$, we obtain

$$\begin{aligned}
b_{i,t} \equiv \langle v_{i,t}\rangle - p_0 \;&=\; q_i \sum_{k=1}^{\infty}(p_k - p_0) \sum_{n=t+(k-1)T}^{t+kT-1} (1-q_i)^n && (40)\\
&=\; q_i (1-q_i)^t \sum_{k=0}^{\infty}(p_{k+1}-p_0)(1-q_i)^{Tk} \sum_{n=0}^{T-1}(1-q_i)^n && (41)\\
&=\; (1-q_i)^t \left[1-(1-q_i)^T\right] \sum_{k=0}^{\infty}(p_{k+1}-p_0)(1-q_i)^{Tk} && (42)\\
&=\; (1-q_i)^t \frac{\sum_{k=0}^{\infty}(1-q_i)^{Tk}(p_{k+1}-p_0)}{\sum_{k=0}^{\infty}(1-q_i)^{Tk}} && (43)\\
&=\; -(1-q_i)^t \delta p_0 + (1-q_i)^t \frac{\sum_{k=0}^{\infty}(1-q_i)^{Tk}\delta p_{k+1}}{\sum_{k=0}^{\infty}(1-q_i)^{Tk}} && (44)\\
&=\; -(1-q_i)^t \delta p_0 + (1-q_i)^t \left[1-(1-q_i)^T\right] \sum_{k=0}^{\infty}(1-q_i)^{Tk}\delta p_{k+1} && (45)
\end{aligned}$$

where we defined

$$\delta p_k \equiv p_k - [p]_\pi \,. \tag{46}$$

Note that we can write the bias, Eq. (45), in the form

$$b_t \equiv \langle v_t\rangle - p_0 = \sum_{k=0}^{\infty} A_k(t)\, \delta p_k \tag{47}$$

where we defined

$$\begin{aligned}
A_0(t) \;&\equiv\; -\left[(1-w_{Slow})(1-q_1)^t + w_{Slow}(1-q_2)^t\right] && (48)\\
A_k(t) \;&\equiv\; (1-w_{Slow})B_{1,k}(t) + w_{Slow}B_{2,k}(t) \qquad (k>0). && (49)
\end{aligned}$$

and

$$B_{i,k}(t) \equiv (1-q_i)^t \left[1-(1-q_i)^T\right](1-q_i)^{T(k-1)} \qquad (k>0, \quad i=1,2). \tag{50}$$

The bias squared is then given by

$$b_t^2 = \sum_{k=0}^{\infty}\sum_{j=0}^{\infty} A_k(t)\, A_j(t)\, \delta p_k \delta p_j = \sum_{k=0}^{\infty} A_k^2(t)\, \delta p_k^2 + \sum_{k\neq j} A_k(t)\, A_j(t)\, \delta p_k \delta p_j. \tag{51}$$

Since δp_k are zero-mean independent variables, averaging over them kills the second, off-diagonal term (this is true even if we don't average over p_0) in the above expression. The bias squared averaged over p_k in the previous blocks (but not on p_0) is thus given by

$$\left[b_t^2|p_0\right]_\pi = \delta p_0^2 A_0^2(t) + \left[\delta p^2\right]_\pi \sum_{k=1}^{\infty} A_k^2(t) \tag{52}$$

$$= (p_0 - [p]_\pi)^2 \left[(1-w_{Slow})(1-q_1)^t + w_{Slow}(1-q_2)^t\right]^2 \tag{53}$$

$$+ \left[\delta p^2\right]_\pi \sum_{k=1}^{\infty} \left[(1-w_{Slow})^2 B_{1,k}^2(t) + w_{Slow}^2 B_{2,k}^2(t) + 2w_{Slow}(1-w_{Slow}) B_{1,k}(t) B_{2,k}(t)\right] \tag{54}$$

Now we have

$$\sum_{k=1}^{\infty} B_{i,k}^2(t) = (1-q_i)^{2t}\left[1-(1-q_i)^T\right]^2 \sum_{k=0}^{\infty}(1-q_i)^{2Tk} \tag{55}$$

$$= (1-q_i)^{2t} \frac{\left[1-(1-q_i)^T\right]^2}{1-(1-q_i)^{2T}} \tag{56}$$

$$= (1-q_i)^{2t} \frac{1-(1-q_i)^T}{1+(1-q_i)^T} \tag{57}$$

and (using $1-Q \equiv (1-q_1)(1-q_2)$)

$$\sum_{k=1}^{\infty} B_{1,k}(t) B_{2,k}(t) = (1-Q)^t \left[1-(1-q_1)^T\right]\left[1-(1-q_2)^T\right] \sum_{k=0}^{\infty}(1-Q)^{Tk} \tag{58}$$

$$= (1-Q)^t \frac{\left[1-(1-q_1)^T\right]\left[1-(1-q_2)^T\right]}{1-(1-q_1)^T(1-q_2)^T} \tag{59}$$

yielding

$$\left[b_t^2|p_0\right]_\pi = (p_0 - [p]_\pi)^2 \left[(1-w_{Slow})(1-q_1)^t + w_{Slow}(1-q_2)^t\right]^2 \tag{60}$$

$$+ \left[\delta p^2\right]_\pi \left[(1-w_{Slow})^2 \frac{1-(1-q_1)^T}{1+(1-q_1)^T}(1-q_1)^{2t} + w_{Slow}^2 \frac{1-(1-q_2)^T}{1+(1-q_2)^T}(1-q_2)^{2t}\right. \tag{61}$$

$$\left. +2w_{Slow}(1-w_{Slow}) \frac{\left[1-(1-q_1)^T\right]\left[1-(1-q_2)^T\right]}{1-(1-q_1)^T(1-q_2)^T}(1-Q)^t\right], \tag{62}$$

for the transient behavior of conditional average bias squared in the current block.

Averaging over p_0 yields

$$\left[b_t^2\right]_\pi = 2\left[\delta p^2\right]_\pi \Bigg[(1-w_{Slow})^2 \frac{1}{1+(1-q_1)^T}(1-q_1)^{2t} + w_{Slow}^2 \frac{1}{1+(1-q_2)^T}(1-q_2)^{2t} \quad (63)$$

$$+2w_{Slow}(1-w_{Slow})\frac{1-\frac{(1-q_1)^T+(1-q_2)^T}{2}}{1-(1-q_1)^T(1-q_2)^T}(1-q_1)^t(1-q_2)^t\Bigg]. \quad (64)$$

Finally, to average over t ranging over the block, we use

$$\frac{1}{T}\sum_{t=1}^{T}(1-q_i)^{2t} = \frac{(1-q_i)^2}{T}\frac{1-(1-q_i)^{2T}}{1-(1-q_i)^2} = \frac{1-(1-q_i)^{2T}}{T\left[(1-q_i)^{-2}-1\right]} \quad (65)$$

$$\frac{1}{T}\sum_{t=1}^{T}(1-Q)^{t} = \frac{1-Q}{T}\frac{1-(1-Q)^{T}}{1-(1-Q)} = \frac{1-(1-Q)^{T}}{T\left[(1-Q)^{-1}-1\right]} \quad (66)$$

to obtain

$$\overline{\left[b_t^2\right]_\pi} = \left[\delta p^2\right]_\pi \Bigg[(1-w_{Slow})^2 \frac{1-(1-q_1)^T}{\frac{T}{2}\left[(1-q_1)^{-2}-1\right]} + w_{Slow}^2 \frac{1-(1-q_2)^T}{\frac{T}{2}\left[(1-q_2)^{-2}-1\right]}$$
$$+2w_{Slow}(1-w_{Slow})\frac{2-(1-q_1)^T-(1-q_2)^T}{T\left[(1-Q)^{-1}-1\right]}\Bigg]. \quad (67)$$

In the regime where $q_2 \ll T^{-1} \ll q_1 \ll 1$ (or $\tau_2 \gg T \gg \tau_1 \gg 1$), we have approximately $(1-q_1)^T \approx 0$, $(1-q_2)^T \approx 1-q_2T$ and $\left[(1-q_i)^{-2}-1\right] \approx 2q_i$ and $\left[(1-Q)^{-1}-1\right] \approx q_1+q_2$, yielding

$$\overline{\left[b_t^2\right]_\pi} = \left[\delta p^2\right]_\pi \left[(1-w_{Slow})^2\frac{\tau_1}{T} + w_{Slow}^2 + 2w_{Slow}(1-w_{Slow})\left(\frac{\tau_1}{T}+\frac{\tau_1}{\tau_2}\right)\right], \quad (68)$$

and in the limit $\tau_2, T \to \infty$:

$$\overline{\left[b_t^2\right]_\pi} = \left[\delta p^2\right]_\pi w_{Slow}^2. \quad (69)$$

Average squared error, optimal w_{Slow}, and undermatching

The long-run average squared error is the sum of average variance and average bias squared and thus from Eqs. (27) and (67) is given by

$$\overline{\left[\langle(v_t-p_0)^2\rangle\right]_\pi} = \overline{\left[\langle\delta v_t^2\rangle\right]_\pi} + \overline{\left[b_t^2\right]_\pi} = C_1(1-w_{Slow})^2 + C_2w_{Slow}^2 + 2C_3w_{Slow}(1-w_{Slow}) \quad (70)$$

where we defined

$$C_1 = \frac{[p(1-p)]_\pi}{2\tau_1-1} + \left[\delta p^2\right]_\pi \frac{1-(1-q_1)^T}{\frac{T}{2}\left[(1-q_1)^{-2}-1\right]} \quad (71)$$

$$C_2 = \frac{[p(1-p)]_\pi}{2\tau_2-1} + \left[\delta p^2\right]_\pi \frac{1-(1-q_2)^T}{\frac{T}{2}\left[(1-q_2)^{-2}-1\right]} \quad (72)$$

$$C_3 = \frac{[p(1-p)]_\pi}{\tau_1+\tau_2-1} + \left[\delta p^2\right]_\pi \frac{2-(1-q_1)^T-(1-q_2)^T}{T\left[(1-q_1)^{-1}(1-q_2)^{-1}-1\right]}. \quad (73)$$

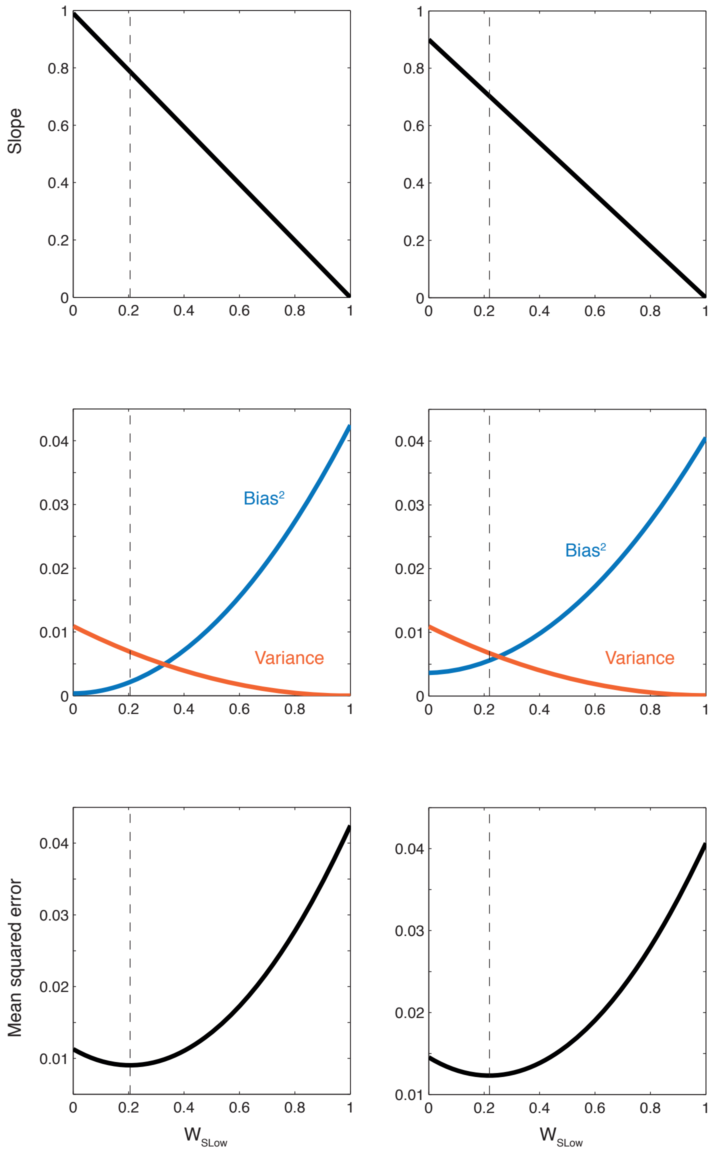

Figure 1: Plots of analytical results: block-averaged undermatching slope, Eq. (94) (top panels), variance Eq. (27) and average squared bias Eq. (67) (middle panels) and average squared error Eq. (70) (bottom panels). For the plots on the left, we used $\tau_1 = 10$, $\tau_2 = 100000$, and $T = 1000$, and for those on the right we used $\tau_1 = 10$, $\tau_2 = 1000$ and $T = 100$. The dashed lines show the optimal weight w_{Slow^*}, Eq. (78), in each case. Here we took the distribution $\pi(p)$ to be the uniform distribution on $[0.1, 0.9]$, yielding $[p]_\pi = \frac{1}{2}$ and $\left[\delta p^2\right]_\pi \simeq 0.0427$ (choosing a uniform distribution on $[0, 1]$ instead, would have yielded $\left[\delta p^2\right]_\pi = \frac{1}{12} \simeq 0.0833$, thus putting more emphasis on squared bias and shifting the minimum of bottom plots, i.e. the optimal w_{Slow}, further to the left).

Here, in each line the first term is the contribution of the variance and the second is the contribution of the average squared bias. Note that in general

$$[p(1-p)]_\pi = [p]_\pi \left(1 - [p]_\pi\right) - \left[\delta p^2\right]_\pi . \tag{74}$$

In particular, for the case where $\pi(\cdot)$ is the uniform distribution on $[0, 1]$, we have

$$\left[\delta p^2\right]_\pi = \left[(p - \frac{1}{2})^2\right]_\pi = \frac{1}{12} \tag{75}$$

$$[p(1-p)]_\pi = \frac{1}{4} - \left[\delta p^2\right]_\pi = \frac{1}{6}. \tag{76}$$

To find the optimal w_{Slow} we have to set the derivative of Eq. (70) w.r.t. w_{Slow} to zero. The latter is proportional to

$$C_1(w_{Slow} - 1) + C_2 w_{Slow} + C_3(1 - 2w_{Slow}), \tag{77}$$

and setting it equal to zero yields

$$w_{Slow^*} = \frac{C_1 - C_3}{C_1 + C_2 - 2C_3}. \tag{78}$$

We can use Eq. (68), to simplify Eq. (71) in the regime $q_2 \ll T^{-1} \ll q_1 \ll 1$ (or $\tau_2 \gg T \gg \tau_1 \gg 1$), obtaining[1]

$$C_1 \approx \frac{[p(1-p)]_\pi}{2\tau_1 - 1} + \left[\delta p^2\right]_\pi \frac{\tau_1}{T} \tag{79}$$

$$C_2 \approx \frac{[p(1-p)]_\pi}{2\tau_2 - 1} + \left[\delta p^2\right]_\pi \tag{80}$$

$$C_3 \approx \frac{[p(1-p)]_\pi}{\tau_1 + \tau_2 - 1} + \left[\delta p^2\right]_\pi \left(\frac{\tau_1}{T} + \frac{\tau_1}{\tau_2}\right). \tag{81}$$

We see that the largest contribution to average error, which is $O(1)$, comes from the bias squared contributed by the slow time scale (the second term in C_2). After that we have the contribution of the fast time scale to variance (first term in C_1) which is $O({\tau_1}^{-1})$ and smaller. For this reason, for realistic underlying time-scales, the optimal w_{Slow}'s will turn out to mainly optimize the squared bias, and hence will be small.

It is much easier to derive these results in the extreme limit $\tau_2, T \to \infty$ (keeping $\tau_2 \gg T$). Firstly, in this case, given that v_2 is a very long-term average of s_t, its value is always very close to the long term average of p, i.e. $[p]_\pi$, with small fluctuations, δv_2, of the order of $1/\sqrt{\tau_2}$. Thus we can ignore the latter and safely write

$$v_{2,t} \approx [p]_\pi . \tag{82}$$

In particular, it is only $v_{1,t}$ which contributes to the variance:

$$\left\langle \delta v_t^2 \right\rangle \approx (1 - w_{Slow})^2 \left\langle \delta v_{1,t}^2 \right\rangle . \tag{83}$$

[1] To be really consistent in the approximations, the first terms on the rights sides of Eq. (79) must also be expanded.

Furthermore, given that $\tau_1 \ll T$, the main contribution to the averages of $\langle v_{1,t}\rangle$ or $\left\langle \delta v_{1,t}^2 \right\rangle$ over t running from $1:T$ comes from t's within the current block that are much larger than τ_1 (i.e., we can ignore the transient behavior of $v_{1,t}$ at the beginning of the block and only consider its steady-state behavior). This means that in Eqs. (22) and (37), we can safely replace $p_{k(t-n)}$ with p_0, the head probability in the current block. The geometric series thus become infinite and we obtain

$$\left\langle \delta v_{1,t}^2 \right\rangle \approx q_1^2 \sum_{n=0}^{\infty} (1-q_1)^{2n}\, p_0(1-p_0) = \frac{q_1^2}{1-(1-q_1)^2} p_0(1-p_0) = \frac{p_0(1-p_0)}{2\tau_1 - 1}. \quad (84)$$

$$\langle v_{1,t}\rangle \approx q_1 \sum_{n=0}^{\infty} (1-q_1)^{n}\, p_0 = p_0 \quad (85)$$

Averaging Eq. (84) over π, and using Eq. (83) we obtain

$$\left[\left\langle \delta v_t^2 \right\rangle\right]_\pi \approx (1-w_{Slow})^2 \frac{[p(1-p)]_\pi}{2\tau_1 - 1}. \quad (86)$$

For the full bias we have $b_t = \langle v_t\rangle - p_0 = (1-w_{Slow})\langle v_{1,t}\rangle + w_{Slow}\langle v_{2,t}\rangle - p_0$, which by Eq. (82) and (85), yields $b_t = w_{Slow}([p]_\pi - p_0) = -w_{Slow}\delta p_0$ (this yields $(1-w_{Slow})$ for the undermatching slope, as an approximation to Eq. (94)). Thus

$$\left[b_t^2\right]_\pi \approx w_{Slow}^2 \left[\delta p^2\right]_\pi . \quad (87)$$

Finally for the average square error we obtain Eq. (70) with

$$C_1 \approx \frac{[p(1-p)]_\pi}{2\tau_1 - 1} \quad (88)$$

$$C_2 \approx \left[\delta p^2\right]_\pi \quad (89)$$

$$C_3 \approx 0. \quad (90)$$

Time-dependent undermatching slope

Going back to Eq. (45) for the bias, since the second term in Eq. (45) vanishes after averaging over p_{k+1}, for the transient of bias conditional on p_0 but averaged over p_k in past blocks we obtain

$$[v_t - p_0|p_0]_\pi = -(p_0 - [p]_\pi)\left[(1-w_{Slow})(1-q_1)^t + w_{Slow}(1-q_2)^t\right]. \quad (91)$$

which we can also write in a form corresponding to the slop of the matching law plot

$$\frac{[v_t|p_0]_\pi - [p]_\pi}{p_0 - [p]_\pi} = 1 - (1-w_{Slow})(1-q_1)^t - w_{Slow}(1-q_2)^t. \quad (92)$$

(assuming a symmetric distribution $\pi(\cdot)$, $[p]_\pi = \frac{1}{2}$). In particular, when $\tau_2 \gg T$ (or $q_2 T \ll 1$) $(1-q_2)^t$ remains approximately equal to unity even for $t = T$ (at the end of the block). Thus we have

$$\frac{[v_t|p_0]_\pi - [p]_\pi}{p_0 - [p]_\pi} = (1-w_{Slow})\left[1-(1-q_1)^t\right], \qquad (\tau_2 \gg T). \quad (93)$$

This shows that there is more undermatching at the beginning of the block, than at the end (where $(1-q_1)^t \ll 1$, if $\tau_1 \ll T$). If we average this over the whole block we obtain for the block-averaged (under)matching slope:

$$\frac{\overline{[v_t|p_0]_\pi} - [p]_\pi}{p_0 - [p]_\pi} = (1 - w_{Slow}) \left[1 - \frac{1-(1-q_1)^T}{Tq_1}\right], \qquad (\tau_2 \gg T) \tag{94}$$

$$\approx (1 - w_{Slow}) \left[1 - \frac{\tau_1}{T}\right], \qquad (\tau_2 \gg T \gg \tau_1) \tag{95}$$

Supplementary Figures

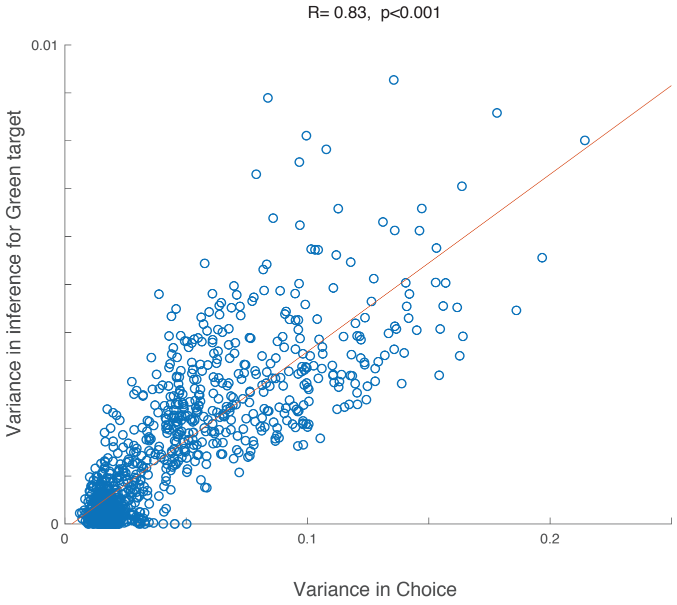

Figure 2: The variance of inference is positively correlated with the variance of choice in our model that was simulated in the actual experimental conditions. The y-axis shows the variance in inference of the Green target, while the x axis shows the variance of choice. The data was generated from the local matching model with a single learning rate [18], where the learning rate was estimated from behavioral data of monkey F on each experimental session. The model was simulated on the same experimental schedule as Monkey F for all sessions, over five times. Each data point was estimated from each session of one simulation. The variance of a signal was defined by the variance of a signal around the low-pass filtered signal, as we described in the Methods section.

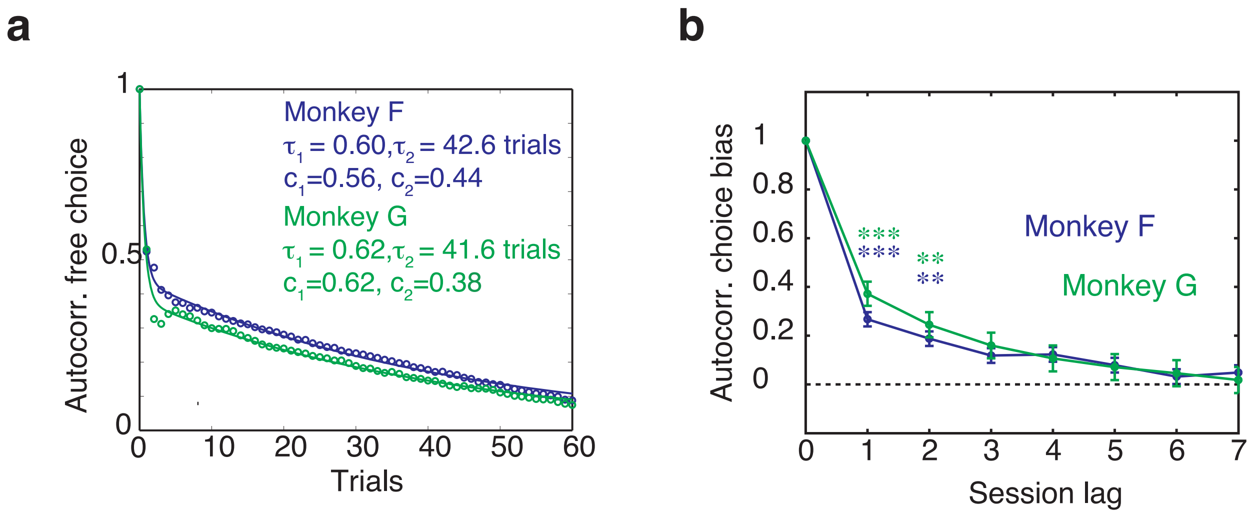

Figure 3: (**a**). Autocorrelation of choice history decays on multiple timescales within a block size. The solid lines indicate the fitting results with a sum of two exponents with relative weights c_1, c_2 and time constants τ_1, τ_1. Those choices reinforced by COD are excluded. (**b**) Both monkeys' session to session color choice bias show significant autocorrelations across different session lags. The stars indicate the significance.

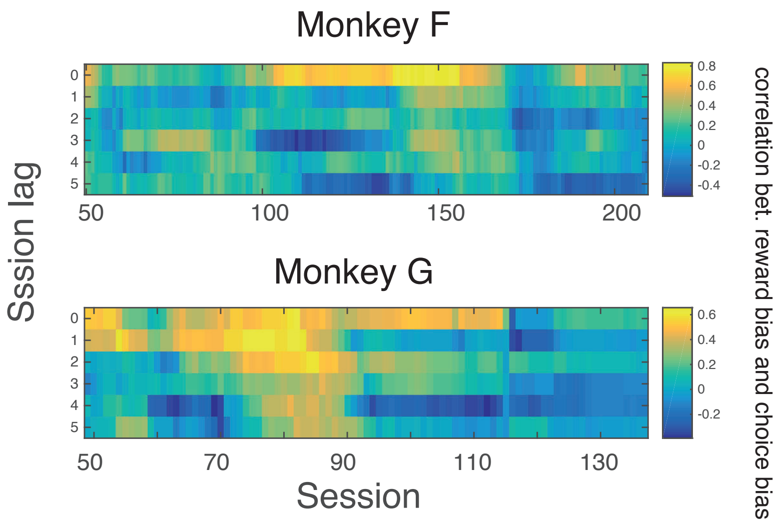

Figure 4: The correlation between color reward imbalance and color choice bias over sliding session windows.

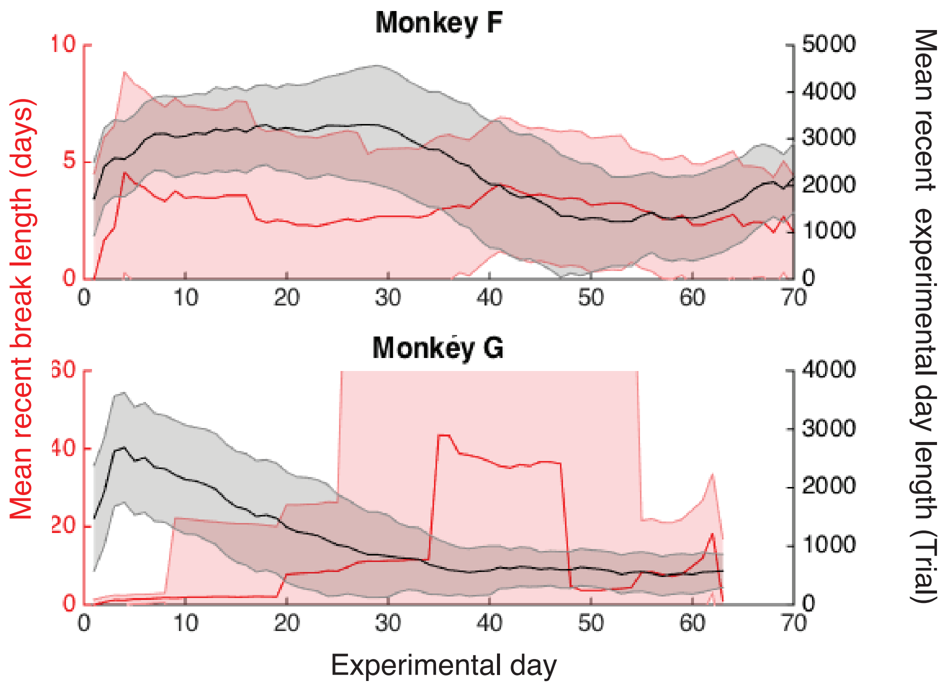

Figure 5: Changes in recent break length (red) and recent experimental day length (black) over the course of experiments.

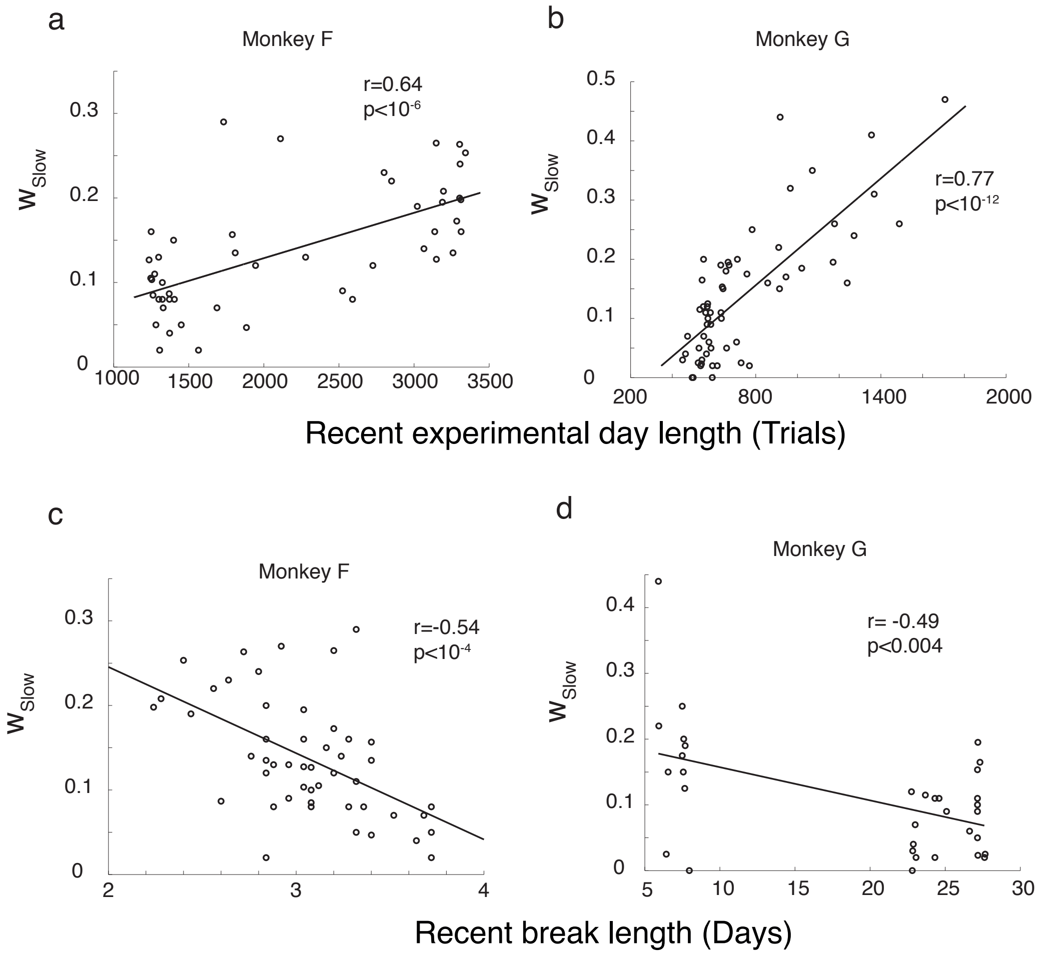

Figure 6: Non monotonic changes in the weight w_{Slow} of the long timescale τ_{Slow} reflect the experimental schedule on a long timescale. **a,b**, The weight of long timescale w_{Slow} correlates with the duration of recent experiments. Daily estimations of w_{Slow} are plotted against the mean length of recent experiments. The weight of the long timescale w_{Slow} is larger when the animal constantly experienced longer experimental sessions. The mean is taken over 18 experimental days (Monkey F) and 12 experimental days (Monkey G), respectively, as they give the largest correlations. **c,d**, The weight of long timescale w_{Slow} anti-correlates with the mean recent inter-experimental-intervals. The weight of the long timescale w_{Slow} is smaller when the animal constantly had long inter-experimental-periods. Daily estimation of w_{Slow} is plotted against the mean recent inter-experimental-intervals. The mean is taken over 25 experimental days (Monkey F) and 32 experimental days (Monkey G), respectively, as they give the largest magnitude of correlations.

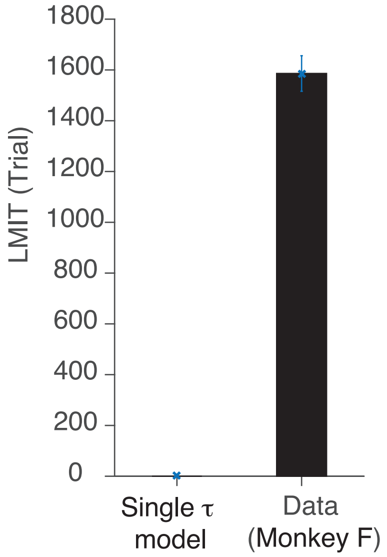

Figure 7: Variable-single-timescale model dose not capture the experimental data. We fitted a local matching model with a single timescale [18] to the data (Monkey F's data). Using the fitted parameter (τ), we simulated the model to generate choice behaviors, which was then used to estimate the LMIT in the same manner as the other analysis. We found no LMIT from the single timescale model, because of the lack of a slow learning.

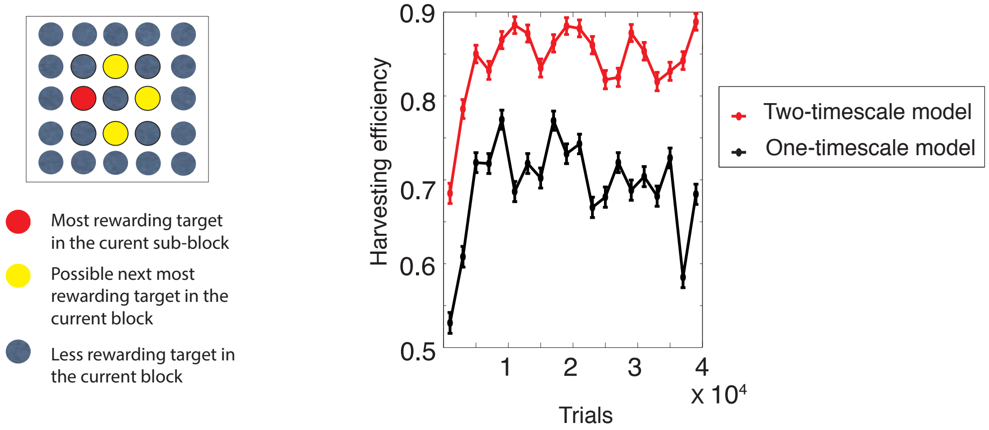

Figure 8: The multi-timescale model performs significantly better in a multi-armed bandit task with a hierarchical block structure. Subjects need to choose a target from 25 different options (colors are not shown to subjects). There is one target that is most rewarding in the current sub-block of trials (red), and the most rewarding target would change among the hot spots (yellow) which are fixed for the current block of trials. Crucially, the most rewarding target changes on a shorter timescale (e.g. every 20 trials), while the hot spots change on a longer timescale (e.g. every 200 trials). In such a situation, learning over both short and long timescales is significantly beneficial.
